# A new diagnostic prediction model for infective endocarditis based on the 2023 duke–international society for cardiovascular infectious disease criteria: a multicenter observational study

**DOI:** 10.1186/s12872-026-05742-8

**Published:** 2026-03-16

**Authors:** Shun Yamashita, Ryosuke Osako, Takanori Yamaguchi, Yoshinori Tokushima, Yukinori Harada, Taiju Miyagami, Yosuke Sasaki, Yasutomo Oda, Masaki Tago

**Affiliations:** 1 Department of General Medicine, Saga University Hospital, 5-1-1 Nabeshima, Saga, 849-8501 Japan; 2 Education and Research Center for Community Medicine, Faculty of Medicine, Saga University, Saga, Japan; 3 Department of Cardiovascular Medicine, Saga University, Saga, Japan; 4 Department of Diagnostic and Generalist Medicine, Dokkyo Medical University, Tochigi, Japan; 5 Department of General Medicine, Faculty of Medicine, Juntendo University, Tokyo, Japan; 6 Department of General Medicine and Emergency Care, Toho University School of Medicine, Tokyo, Japan

**Keywords:** Infective endocarditis, A diagnostic prediction model, Machine learning, 2023 Duke-ISCVID criteria, Objective variables

## Abstract

**Background:**

Infective endocarditis (IE) remains a diagnostically challenging disease with diverse symptoms. Whether IE is suspected frequently depends on clinical judgement. A reliable model to estimate the likelihood of IE at presentation across clinical departments is needed; however, no such model is currently available. We aimed to develop such a diagnostic prediction model using objective and reproducible variables and to validate its performance.

**Methods:**

We included inpatients aged ≥ 20 years who had either a diagnosis code for IE or for undiagnosed fever during hospitalization. The model was developed using data from a single university hospital between 2007 and 2017 (derivation cohort) and validated with data from four university hospitals between 2018 and 2020 (validation cohort). IE was diagnosed according to the 2023 Duke-International Society for Cardiovascular Infectious Diseases (ISCVID) criteria. Variables were selected using the Boruta algorithm and Least Absolute Shrinkage and Selection Operator regression. Multivariable logistic regression analysis was used to estimate odds ratios and 95% confidence intervals and to construct the model. Model performance was assessed in both cohorts using the area under the curve (AUC). In the validation cohort, calibration was also assessed using the calibration slope, Hosmer–Lemeshow test, and stratified likelihood ratio.

**Results:**

The derivation and validation cohorts included 105 (46 IE) and 286 (106 IE) patients, respectively. Predictors in the final model were the presence of a cardiac murmur, log-transformed platelet count, neutrophil percentage, presence of pleural effusion, and a quick sequential [sepsis-related] organ failure assessment score ≥ 2. The AUC was 0.918 in the derivation cohort. In the validation cohort, the AUC was 0.859, with a Hosmer– Lemeshow test p-value of 0.246 and a calibration slope of 0.759. The stratified likelihood ratio ranged from 0.04 to 9.71 and increased with higher model scores.

**Conclusions:**

This model showed high discrimination and good calibration using objective variables that are readily available early after admission. Furthermore, this is the first model to predict IE based on the 2023 Duke-ISCVID criteria. Further multicenter validation in community hospitals would enhance generalizability.

**Supplementary Information:**

The online version contains supplementary material available at 10.1186/s12872-026-05742-8.

## Introduction

Infective endocarditis (IE) is a diagnostically challenging disease with diverse symptoms, including fever, dyspnea, joint pain, limb paralysis, and altered consciousness, and carries a high mortality rate of 11–30% [1–5]. Because of its heterogeneous clinical presentation, patients with IE may initially seek care in various medical specialties, including orthopedics, neurosurgery, dermatology, or other non-infectious disease departments [1]. Diagnosis requires advanced investigations such as multiple blood cultures, echocardiography, contrast-enhanced computed tomography (CT), or magnetic resonance imaging (MRI). 18 F-Fluoro-D-glucose positron emission tomography/CT may be required for IE involving prosthetic valves [6]. However, the decision to suspect IE and order these tests often depends on the physician’s clinical judgment. If IE is not suspected, antibiotics may be started before blood cultures are obtained, complicating diagnosis and worsening prognosis [7, 8]. Therefore, a diagnostic prediction model that allows early estimation of IE risk across departments could help reduce inappropriate antibiotic use before blood cultures are collected.

Several diagnostic prediction models for IE have been developed. However, most require prior identification of causative organisms, such as *Staphylococcus aureus*, *Enterococcus* spp., or *Streptococcus* spp., via blood cultures [9–11]. These models are less useful given the delay of several days required for blood culture results, and do not adequately address the initial diagnostic challenge. To overcome this limitation, three models independent of blood culture results have been proposed: (1) a U.S. multicenter model for intravenous drug use-associated IE [12, 13], (2) an Italian single-center emergency department model [14], and (3) a Japanese multicenter model developed and validated by our group for hospitalized patients across all clinical departments [5, 15]. Each has limitations despite favorable area under the curve (AUC) values. The first may be overfitted because of its small sample size [12, 13]. The second was developed in a single emergency department, included 12 predictors, and relied on a subjective variable: high suspicion of IE [14]. The third could predict IE across all departments using five readily available variables, including transfer by ambulance, the presence of a cardiac murmur, the presence of a pleural effusion, neutrophil percentage, and platelet count. However, its performance may vary across patient populations and health care systems because “transfer by ambulance” is inconsistent. Ambulances are sometimes used for non-emergency reasons, including health-related anxiety, lack of awareness of alternative care, or preference for the emergency department [5, 16]. Furthermore, most existing models were based on the modified Duke criteria, which show limited accuracy for prosthetic valve endocarditis [17].

To the best of our knowledge, no prediction model has yet been reported for IE diagnosed using the newly proposed 2023 Duke-International Society for Cardiovascular Infectious Diseases (ISCVID) criteria [6]. In this study, we aimed to identify objective predictors beyond those included in our previous model [5, 15] and develop a new diagnostic prediction model for IE based on the 2023 Duke-ISCVID criteria in patients with fever of undetermined origin despite standard diagnostic evaluation, including urinalysis, chest radiography, and blood tests. We further validated the model using the cohort from our previous validation study [5].

## Materials and methods

### Study design

We included patients aged ≥ 20 years who were assigned International Statistical Classification of Diseases and Related Health Problems-10th Revision (ICD-10) code I33.0 (IE) or R50.9 (undiagnosed fever) during hospitalization within the study period. The study comprised two parts: model derivation and validation. In the first part, we retrospectively reviewed data of hospitalized patients from all clinical departments at Saga University Hospital between September 2007 and December 2017, where we developed a diagnostic prediction model (derivation cohort). In the second part, we retrospectively reviewed data of hospitalized patients from all clinical departments at four university hospitals in Japan between January 2018 and December 2020 to validate this model (validation cohort). The datasets used in the development and validation cohorts of this study were the same as those used in our two previous studies, in which we first developed and validated the model [1, 5]. Exclusion criteria were as follows: (1) patients without fever ≥ 37 °C before the admission decision; (2) patients with I33.0 who had nosocomial fever onset, were referred for valvular surgery after IE treatment at another hospital, or were not classified as “definite” under the 2023 Duke-ISCVID criteria; (3) patients with R50.9 who had a confirmed diagnosis before the admission decision or who lacked chest radiography, blood tests, or urinalysis before the admission decision; and (4) patients with a confirmed non-infectious disease despite being classified as “definite” by the 2023 Duke-ISCVID criteria. In this context, ‘blood tests’ refer to routine laboratory examinations obtained during the outpatient assessment before the admission decision, including a complete blood count and basic biochemical tests, such as C-reactive protein. In the derivation cohort, we excluded patients with missing data for any variables other than those required for the 2023 Duke-ISCVID assessment. In the validation cohort in which the predictors were identified, patients with missing data for any of the model variables were excluded. At each institution, at least two physicians reviewed the medical records and recorded the results of examinations required by the 2023 Duke-ISCVID criteria. Patients meeting the “definite” criteria were assigned to the IE group, and all others to the non-IE group.

### Study settings

The derivation cohort was established at Saga University Hospital, the only university hospital in Saga Prefecture, Northern Kyushu, Japan, with a population of approximately 810,000 individuals. This tertiary care center has 602 inpatient beds and 29 clinical departments, including general medicine, cardiology, cardiovascular surgery, and infectious diseases. The validation cohort included patients from four university hospitals in Japan: Saga University Hospital, Dokkyo University Hospital, Juntendo University Hospital, and Toho University Hospital. Dokkyo University Hospital is located in Tochigi Prefecture, a suburban area in northern Kanto with a population of approximately 1.9 million residents. It has 1,195 inpatient beds. Juntendo University Hospital and Toho University Hospital are in Tokyo, a major metropolitan area with approximately 14.04 million residents. Juntendo University Hospital and Toho University Hospital have 1,036 and 916 inpatient beds, respectively. All three tertiary care centers, each comprising 31 to 34 clinical departments, include internal medicine, cardiology, cardiovascular surgery, and infectious diseases.

### Data sources

The survey items are listed in Supplementary Material Table 1. For patients on long-term antibiotics for chronic diseases (for example, chronic bronchitis), blood cultures obtained after fever onset but before additional antibiotics were considered equivalent to no prior antibiotic exposure. Acute respiratory failure on admission was defined as a peripheral oxygen saturation (SpO₂) ≤ 94% on room air or the need for supplemental oxygen. Altered consciousness was defined as a Japan Coma Scale score ≥ I-1 or a Glasgow Coma Scale score ≤ 14 on the day of admission. The quick sequential [sepsis-related] organ failure assessment (q-SOFA) score was determined based on the following parameters: respiratory rate ≥ 22 breaths/min, systolic blood pressure ≤ 100 mmHg, and altered consciousness, as previously described [18]. One point was assigned for each parameter, yielding a total score ranging from 0 to 3; a score ≥ 2 suggests possible sepsis [18]. The systemic inflammatory response syndrome (SIRS) score was determined based on the following parameters: body temperature < 36 °C or > 38 °C, heart rate ≥ 90 beats/min, respiratory rate ≥ 20 breaths/min or arterial carbon dioxide pressure < 32 mmHg, and white blood cell count ≥ 12 × 10^3^/µL or ≤ 4 × 10^3^/µL [19]. One point was assigned for each parameter, yielding a total score ranging from 0 to 4 [19]. The presence of pulmonary edema or pleural effusion on chest radiography or chest CT was determined by a radiologist’s report. If a radiologist’s report was unavailable, two general physicians independently reviewed the image. When their assessments agreed, the findings were accepted; if they disagreed, the images were re-evaluated jointly, and a consensus was reached.

### Statistical analysis and sample size

In both the derivation and validation cohorts, categorical variables are expressed as percentages and were compared using the chi-square (χ^2^) test. Continuous variables are expressed as medians with interquartile ranges (IQRs) and were compared using the Mann–Whitney *U* test. Statistical significance was set at *p* < 0.05.

Examinations required by the 2023 Duke-ISCVID criteria were performed at the attending physician’s discretion; if an examination was not performed, the result was recorded as “no abnormality.” Right-skewed continuous variables were log-transformed using the natural logarithm. In the derivation cohort, we applied the Boruta algorithm to candidates to identify predictors of definite IE according to the 2023 Duke-ISCVID criteria; “transfer by ambulance” was excluded because of inconsistent recording. Variables confirmed by the Boruta algorithm were then subjected to Least Absolute Shrinkage and Selection Operator (LASSO) regression, and the selected variables were incorporated into the model. This two-step approach screened variables with Boruta, and then used LASSO for regularization and further selection. Details of the LASSO implementation are provided in Supplementary Material Methods 1. We adopted this two-step approach to avoid overfitting and enhance bedside usability by limiting the number of predictors to a practical minimum. Multivariable logistic regression analysis was used to estimate odds ratios and 95% confidence intervals and to construct the model. Model performance was assessed in both cohorts using the area under the curve (AUC). logistic regression analysis was used to estimate odds ratios (OR) and 95% confidence intervals (CI) and to construct the prediction model.

Model performance was assessed in the validation cohort. Discrimination was evaluated using the AUC, and calibration was assessed using the calibration slope and calibration-in-the-large (intercept), Hosmer–Lemeshow test, and stratified likelihood ratio. For cutoff values corresponding to 90% sensitivity, Youden’s Index, and 90% specificity, we calculated the predicted probability of IE, sensitivity, specificity, positive predictive value (PPV), and negative predictive value (NPV).

Based on our previous model of five predictors [5, 15] and the rule of thumb of ≥ 10 outcome events per predictor, we prespecified a minimum of 50 outcome events for the derivation cohort [20]. For the validation cohort, a minimum of 50 cases was prespecified to permit assessment of discrimination using receiver operating characteristic analysis [14].

Analyses were performed using SPSS (version 29; IBM Corp., Armonk, NY, USA) and R. The Boruta package (version 8.0.0) and the caret R package (version 7.0–1) were used for feature selection and LASSO regression, respectively.

### Ethical considerations

This study was approved by the Ethics Committee of Saga University Hospital (file number: 2025-01-R-05) and conducted in accordance with the Ethical Guidelines for Medical and Health Research Involving Human Subjects in Japan and the principles of the Declaration of Helsinki. Eligible participants were informed of the study objectives via the clinical research center website of Saga University Hospital. In accordance with an ethics-committee-approved opt-out procedure, consent was considered to have been obtained unless participants explicitly declined participation. Patient confidentiality was maintained throughout the study. This study was registered with the University Hospital Medical Information Network (UMIN) on July 2, 2025 (www.umin.ac.jp; UMIN ID: UMIN000058353).

## Results

### Allocation and participant characteristics

Participant enrollment and group allocation are shown in Supplementary Material Fig. 1. In the derivation cohort, 106 and 122 cases were identified using ICD-10 codes I33.0 and R50.9, respectively. In the validation cohort, 264 and 348 cases were identified with the same codes. After applying the exclusion criteria, 105 cases (46 IE and 59 non-IE) in the derivation cohort and 286 cases (106 IE and 180 non-IE) in the validation cohort were included in the analysis. Comparison of patient characteristics for both cohorts is presented in Table 1. Compared with the validation cohort, the derivation cohort had more patients who received antibiotics before blood culture collection and fewer who used immunosuppressive agents. No significant differences in other patient characteristics were observed between the cohorts. The distribution of underlying diseases in the non-IE groups of the derivation and validation cohorts is presented in Supplementary Material Table 2.

**Table 1:** Patient characteristics in the derivation and validation cohorts

	Derivation cohort (*N* = 105)	Validation cohort (*N* = 286)	*p*-value
Age	66.0 (55.5–77.0)	69.0 (56.0–79.0)	0.490
Male	59 (56)	156 (55)	0.772
The number of IE patients	46 (44)	106 (37)	0.225
Transfer by ambulance	26 (25)	85 (30)	0.335
Duration of hospital stays	23.0 (12.0–41.5)	21.0 (10.0–46.5)	0.747
Past history			
IE	2 (2)	12 (4)	0.226
Chronic skin disease	12 (11)	22 (8)	0.245
CKD	12 (11)	52 (18)	0.110
DM	19 (18)	54 (19)	0.860
Malignancy	14 (13)	54 (19)	0.200
Use of steroids	8 (8)	22 (8)	0.981
Use of immunosuppressants	3 (3)	26 (9)	0.036
Administration of antibiotics^§^	60 (58)	82 (29)	< 0.001
Outcomes			
Mortality at discharge	13 (12)	24 (8)	0.237
30-day mortality after admission	12/102 (12)	16/272 (6)	0.054

Categorical data are expressed as n (%) and were compared using the χ^2^ test. Continuous variables are expressed as median (interquartile range) and were compared using the Mann–Whitney *U* test

*IE* infective endocarditis, *CKD* chronic kidney disease, *DM* diabetes mellitus

§: prior to obtaining blood culture samples

### Derivation cohort

Patient characteristics in the IE and non-IE groups within the derivation cohort are presented in Table 2. Patients in the IE group had a significantly higher proportion of ambulance transfers and a significantly longer hospital stay than those in the non-IE group. No significant differences were observed between the groups in age, sex, medical history, antibiotic use before blood culture collection, or clinical outcomes. Admission findings are summarized in Table 3. Compared to the non-IE group, the IE group had significantly higher pulse rates and respiratory rates, q-SOFA and SIRS scores, white blood cell count, neutrophil percentage, total bilirubin, and blood urea nitrogen (BUN), and lower platelet count and serum albumin level. The presence of acute respiratory failure, disturbance of consciousness, q-SOFA ≥ 2, cardiac murmur, pulmonary edema, and pleural effusion was significantly higher in the IE group than in the non-IE group.

**Table 2:** Patient characteristics in the IE and non-IE groups in the derivation and validation cohorts

	Derivation cohort			Validation cohort		
	IE (*N* = 46)	Non-IE (*N* = 59)	*p*-value	IE (*N* = 106)	Non-IE (*N* = 180)	*p*-value
Age	70.0 (56.0–75.0)	66.0 (52.0–79.0)	0.526	70.5 (57.0–78.0)	69.0 (52.3–79.0)	0.312
Male	27 (59)	32 (54)	0.648	71 (67)	85 (47)	0.001
Transfer by ambulance	25 (54)	1 (2)	< 0.001	38 (36)	47 (26)	0.082
Duration of hospital stays	38.5 (15.8–57.3)	18.0 (12.0–29.0)	0.002	44.5 (25.0–58.0)	14.0 (9.0–24.0)	< 0.001
Past history						
IE	2 (4)	NA	0.190	11 (10)	1 (1)	< 0.001
Chronic skin disease	5 (11)	7 (12)	0.874	13 (12)	9 (5)	0.026
CKD	7 (15)	5 (8)	0.281	24 (23)	28 (16)	0.133
DM	9 (20)	10 (17)	0.730	19 (18)	35 (19)	0.751
Malignancy	3 (7)	11 (19)	0.070	20 (19)	34 (19)	0.997
Use of steroids	4 (9)	4 (7)	0.496	4 (4)	18 (10)	0.056
Use of immunosuppressants	2 (4)	1 (2)	0.407	7 (7)	19 (11)	0.256
Administration of antibiotics^§^	22 (48)	38 (64)	0.070	20 (19)	62 (34)	0.004
Outcomes						
Mortality at discharge	8 (17)	5 (8)	0.169	19 (18)	5 (3)	< 0.001
30-day in-hospital mortality	8 (17)	4 (7)	0.067	11/104 (11)	5/168 (3)	0.010

Categorical data are expressed as n (%) and were compared using the χ^2^ test. Continuous variables are expressed as median (interquartile range) and were compared using the Mann–Whitney *U* test

*IE* infective endocarditis, *CKD* chronic kidney disease, *DM* diabetes mellitus, *NA* not available

§: prior to obtaining blood culture samples

**Table 3:** Findings on admission in the IE and non-IE groups in the derivation and validation cohorts

	Derivation cohort			Validation cohort		
	IE (*N* = 46)	Non-IE (*N* = 59)	*p*-value	IE (*N* = 106)	Non-IE (*N* = 180)	*p*-value
Vital sign						
Pulse rate (beats/min)	101.0 (86.0–111.3)	94.0 (79.0–108.0)	0.040	89.5 (73.0–108.0)	92.0 (80.0–106.0)	0.824
Respiratory rate (beats/min)	21.5 (16.8–25.0)	18.0 (15.0–20.0)	0.002	20.0 (16.0–24.0)	20.0 (16.0–21.0)	0.037
Acute respiratory failure	23 (50)	11 (19)	< 0.001	43 (41)	51/176 (29)	0.046
Disturbance of consciousness	14 (30)	5 (8)	0.004	29/98 (30)	33/151 (22)	0.168
q-SOFA score	1.0 (0–2.0)	0 (0–1.0)	< 0.001	1.0 (0–1.0)	0 (0–1.0)	0.030
q-SOFA ≥ 2	17 (37)	5 (8)	< 0.001	19 (18)	17 (9)	0.037
SIRS score	2.0 (2.0–3.0)	2.0 (1.0–2.0)	0.003	2.0 (1.0–3.0)	2.0 (1.0–3.0)	0.817
Physical findings						
Cardiac murmur	26 (57)	7 (12)	< 0.001	65 (61)	22 (12)	< 0.001
Chest images						
Pulmonary edema	8 (17)	2 (3)	0.020	15 (14)	7 (4)	0.002
Pleural effusion	29 (63)	18 (27)	< 0.001	41 (39)	41 (23)	0.004
Laboratory findings						
WBC (×10⁹/L)	13.4 (10.0–17.7)	10.3 (7.2–13.4)	0.005	9.5 (7.5–13.4)	8.4 (5.0–12.3)	0.004
Neutrophil percentage (%)	89.2 (79.0–92.7)	81.0 (73.0 − 85.7)	< 0.001	86.7 (81.2–91.4))	78.7 (69.2–86.0)	< 0.001
Platelet count (×10^4^/µL)	11.8 (5.1–22.2)	27.8 (16.1 − 40.8)	< 0.001	15.2 (11.3–21.8)	20.9 (14.5–34.6)	< 0.001
Albumin (g/dL)	2.4 (2.1–3.0)	2.8 (2.4–3.2)	0.007	2.9 (2.5–3.5)	3.1 (2.6–3.6)	0.174
Total bilirubin (µmol/L)	0.9 (0.7–1.4)	0.7 (0.5–1.0)	0.011	0.7 (0.5–1.1)	0.7 (0.4–0.9)	0.324
LDH (IU/L)	316.0 (241.3–416.8)	231.0 (171.0–331.0)	0.008	269.0 (222.5–363.3)	238.0 (180.0–300.3)	< 0.001
BUN (mg/dL)	21.9 (13.0–41.4)	16.1 (11.5–22.8)	0.007	21.0 (13.9–34.7)	15.0 (11.0–22.9)	< 0.001
Creatinine (mg/dL)	0.9 (0.7–1.5)	0.8 (0.60–1.0)	0.077	0.9 (0.8–1.6)	0.8 (0.6–1.1)	0.001
CRP (mg/dL)	13.4 (5.5–18.7)	10.9 (3.9–17.5)	0.163	8.2 (3.5–17.1)	8.4 (3.1–14.1)	0.198

Categorical data are expressed as n (%) and were compared using the χ^2^ test. Continuous variables are expressed as median (interquartile range) and were compared using the Mann–Whitney *U* test

*IE* infective endocarditis, *q-SOFA* quick sequential [sepsis-related] organ failure assessment, *SIRS* systemic inflammatory response syndrome, *WBC* white blood cell count, *LDH* lactate dehydrogenase, *BUN* blood urea nitrogen, *CRP* C-Reactive Protein

### Selection of model variables in the derivation cohort

**Fig. 1:**
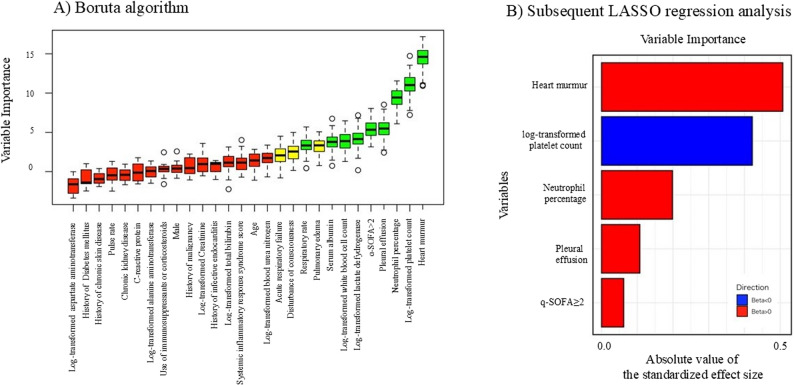
Boruta algorithm and subsequent LASSO regression results in the derivation cohort. **A** Boruta algorithm; **B** Subsequent LASSO regression analysis. The Boruta algorithm identified the following variables as strongly associated with infective endocarditis: presence of a cardiac murmur, log-transformed platelet count, neutrophil percentage, presence of pleural effusion, q-SOFA score ≥2, serum albumin level, and log-transformed serum lactate dehydrogenase level. Subsequent LASSO regression analysis selected five predictors included in the final model: presence of a cardiac murmur, neutrophil percentage, log-transformed platelet count, presence of pleural effusion, and q-SOFA ≥2. LASSO, least absolute shrinkage and selection operator; q-SOFA, quick sequential [sepsis-related] organ failure assessment

Normality of continuous variables was assessed using histograms. Variables such as white blood cell count, platelet count, lactate dehydrogenase (LDH), total bilirubin, aspartate aminotransferase, alanine aminotransferase, BUN, and serum creatinine showed right-skewed distributions and were log-transformed using the natural logarithm. Using the Boruta algorithm, the following variables were identified as highly important: presence of a cardiac murmur, log-transformed platelet count, neutrophil percentage, presence of pleural effusion, q-SOFA ≥ 2, log-transformed serum LDH level, log-transformed white blood cell count, serum albumin level, and respiratory rate (Fig. 1). Subsequently, LASSO regression was performed on these variables, and five predictors were selected for the final model: presence of a cardiac murmur, neutrophil percentage, log-transformed platelet count, presence of pleural effusion, and q-SOFA ≥ 2 (Fig. 1).

### Model performance in the derivation cohort

We conducted multivariable logistic regression using the five selected predictors. The ORs and 95% CIs for each variable are presented in Table 4. Based on the regression coefficients, we constructed the prediction formula as follows:

**Table 4:** Multivariable logistic regression analysis for the five model variables identified in the derivation cohort

	Odds ratio	95%CI	*P* value
Cardiac murmur	19.143	4.795–76.416	< 0.001
q-SOFA ≥ 2	3.353	0.662–16.983	0.144
Pleural effusion	4.230	1.286–13.915	0.018
Neutrophil percentage	1.100	1.031–1.174	0.004
Log-transformed platelet count	0.271	0.122–0.603	< 0.001

*CI* Confidence interval, *q-SOFA* quick sequential [sepsis-related] organ failure assessment

−6.175 + 1.210 × (q-SOFA score: “≥2” = 1, “<2” = 0) + 2.952 × (cardiac murmur: presence = 1, absence = 0) + 1.442 × (pleural effusion: presence = 1, absence = 0) + 0.096 × (neutrophil percentage) − 1.306 × ln[platelet count (×10^4^/µL)].

**Fig. 2:**
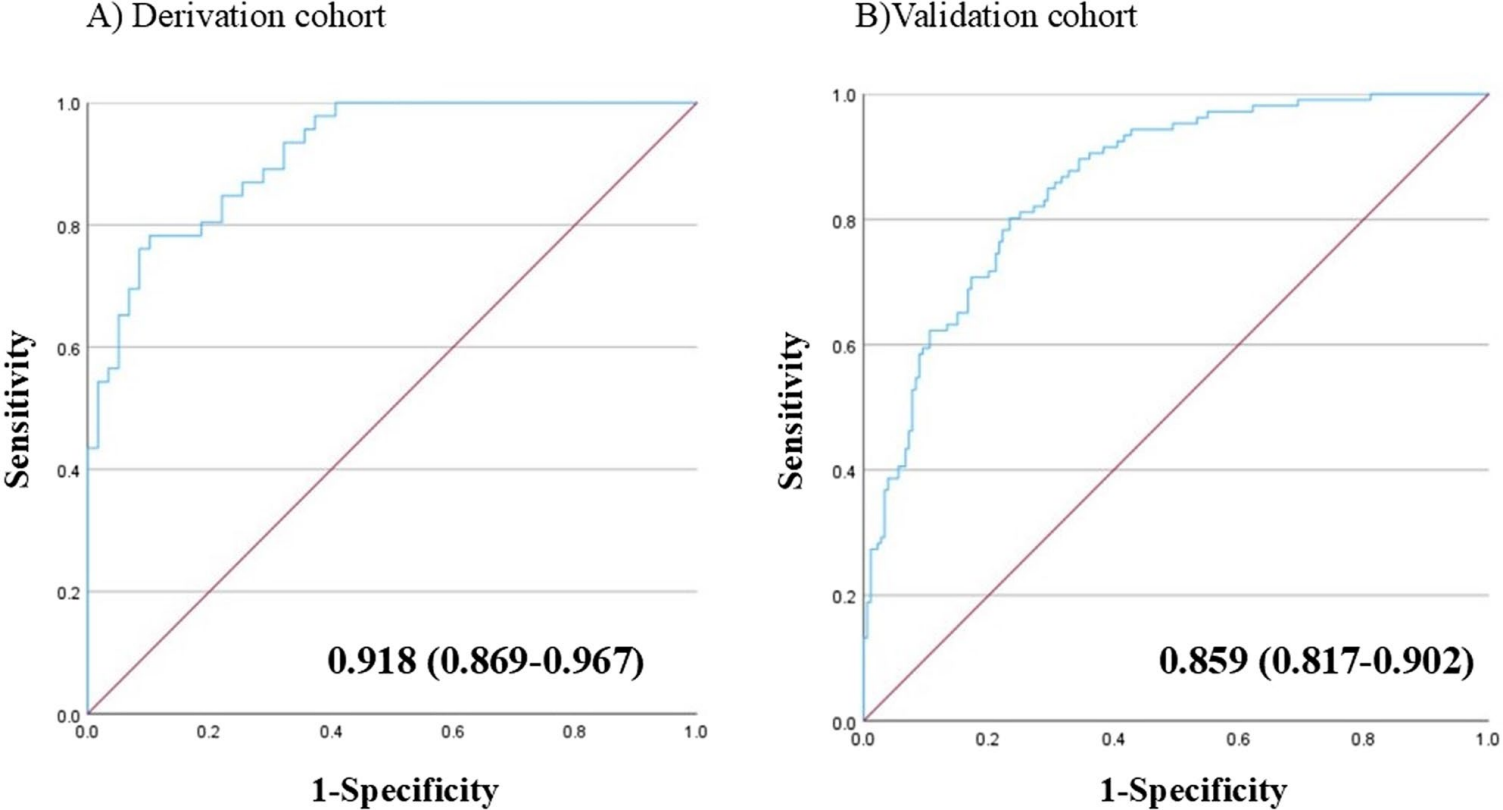
Discriminative ability of the newly developed diagnostic model. **A** AUC in the derivation cohort; **B** AUC in the validation cohort. The newly developed model demonstrated AUCs of 0.918 (95% CI: 0.869–0.967) and 0.859 (95% CI: 0.817–0.902) in the derivation and validation cohorts, respectively AUC, area under the curve; CI, confidence interval

The AUC was 0.918 (95% CI: 0.869–0.967) (Fig. 2).

### Validation cohort

Patient characteristics in the validation cohort are summarized in Table 2. Compared with the non-IE group, the IE group had significantly higher proportions of male patients, histories of IE and chronic skin disease, higher in-hospital and 30-day mortality rates, and longer hospital stays. In contrast, antibiotics were administered less frequently prior to blood culture collection in the IE group. Findings on admission are presented in Table 3. Several variables, including pulse rate, disturbance of consciousness, SIRS score, serum albumin level, and serum total bilirubin level, that differed significantly between the IE and non-IE groups in the derivation cohort, did not differ significantly between groups in the validation cohort.

### Model performance in the validation cohort

**Fig. 3:**
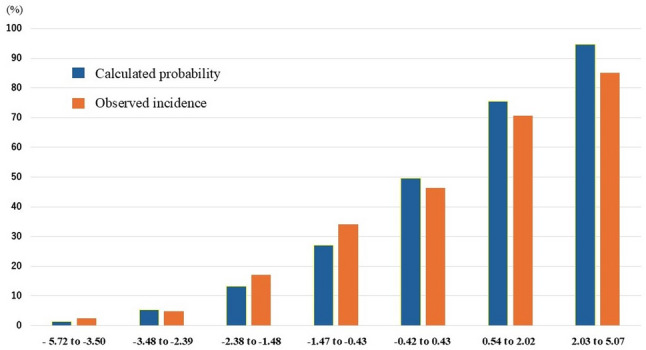
Calibration plot of the newly developed diagnostic model in the validation cohort. In the validation cohort, both the predicted probability and observed incidence increased with higher model scores. The model calibration was well with the Hosmer–Lemeshow test of 0.246 and a calibration slope of 0.759 (standard error 0.092, 95% CI 0.58–0.94, *p* < 0.001)

**Fig. 4:**
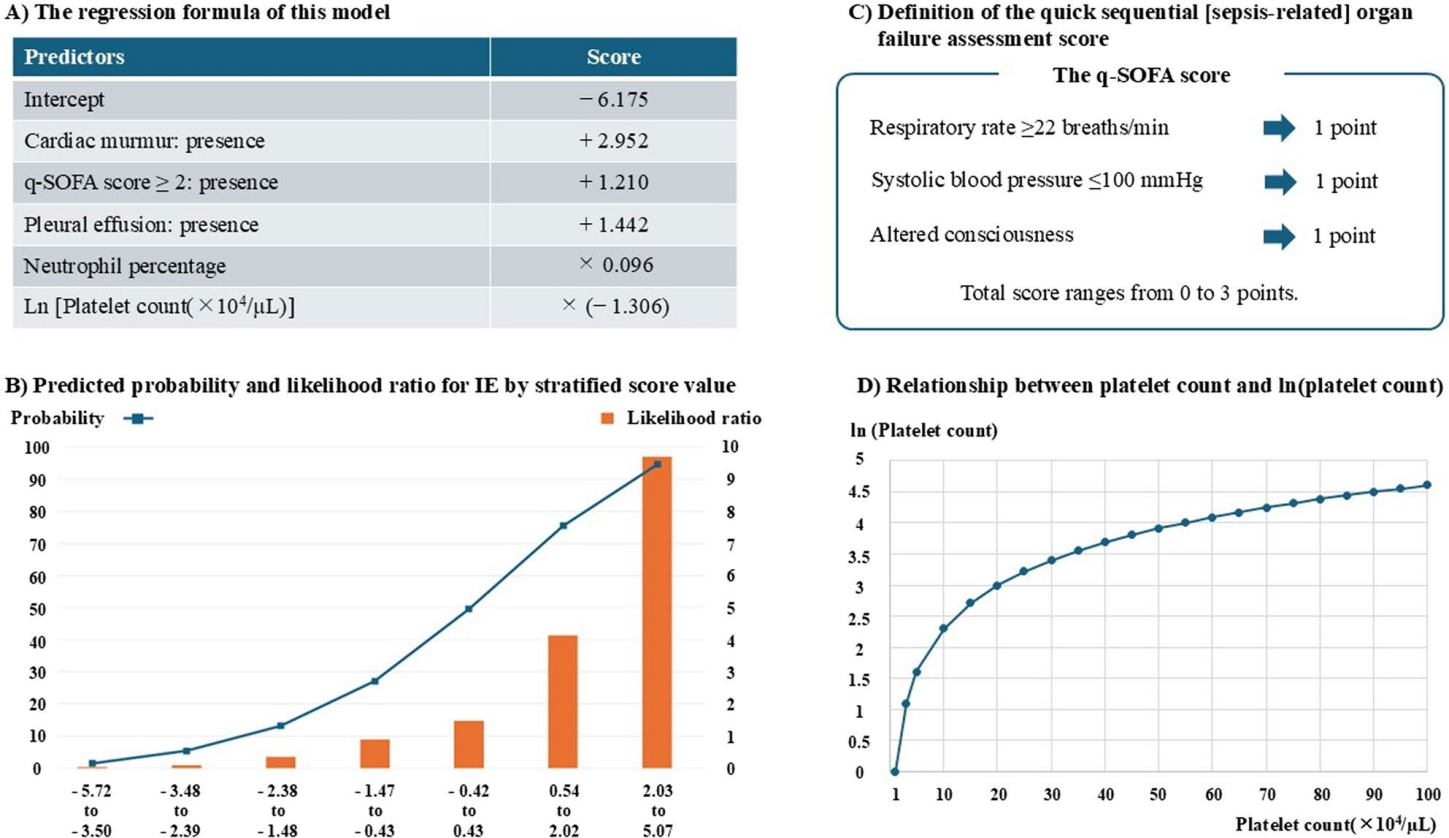
Integrated visualization of model components, coefficients, score-stratified predictive probabilities, and likelihood ratios. **A** The regression formula of this model. **B** Predicted probability and likelihood ratio for IE by stratified score value. **C** Definition of the quick sequential [sepsis-related] organ failure assessment score. **D** Relationship between platelet count and ln(platelet count). The regression formula of this model was constructed as follows: −6.175 + 1.210 × (q-SOFA score: “≥2” = 1, “<2” = 0) + 2.952 × (cardiac murmur: presence = 1, absence = 0) + 1.442 × (pleural effusion: presence = 1, absence = 0) + 0.096 × (neutrophil percentage) − 1.306 × ln[platelet count (×10^4^/µL)] (**A**). The score-stratified predictive probabilities of IE were 1.48% to 94.7% and the stratified likelihood ratios were 0.04 to 9.71. They increased with higher model scores (**B**). The q-SOFA score was assessed using respiratory rate ≥ 22 breaths/min, systolic blood pressure ≤ 100 mmHg, and the presence of altered consciousness. The q-SOFA score is calculated by awarding one point for each component, for a total score ranging from 0 to 3 (**C**). Platelet count showed right-skewed distributions and was log-transformed using the natural logarithm (**D**). IE, infective endocarditis; q-SOFA, quick sequential [sepsis-related] organ failure assessment

In the validation cohort, the model showed discrimination, with an AUC of 0.859 (95% CI: 0.817–0.902) (Fig. 2). Calibration revealed a calibration slope of 0.759 (standard error 0.092, 95% CI 0.58–0.94, *p* < 0.001) and a calibration-in-the-large (intercept) of − 0.146 (standard error 0.158, 95% CI − 0.46 to 0.16, *p* = 0.356), with a Hosmer–Lemeshow test p-value of 0.246 (Fig. 3). Figure 4 shows the regression coefficients of the model components, predictive probabilities stratified by score, and the corresponding likelihood ratios. The stratified likelihood ratio ranged from 0.04 to 9.71 and increased with higher model scores. Cutoffs of − 1.40, − 0.61, and 0.48 were chosen to yield 90% sensitivity, to maximize Youden’s index, and to yield 90% specificity, respectively. The PPV and NPV for these cutoffs were 59% and 91%, 67% and 88%, and 78% and 79%, respectively (Table 5).

**Table 5:** The cut-off points determined in the present study and the original study

Statistics for 3 cutoff points	Derivation cohort	Validation cohort
Sensitivity around 90%		
Cutoff value for scores	-1.06	-1.40
Probability^†^	26	20
Sensitivity	89	90
Specificity	71	64
Positive predictive value	71	59
Negative predictive value	89	91
Youden’s Index		
Cutoff value for scores	0.24	-0.61
Probability^†^	56	35
Sensitivity	78	80
Specificity	89	77
Positive predictive value	86	67
Negative predictive value	84	88
Specificity around 90%		
Cutoff value for scores	0.26	0.49
Probability^†^	57	62
Sensitivity	76	59
Specificity	90	90
Positive predictive value	85	78
Negative predictive value	83	79

†: Predicted probability of IE at cutoff values of the prediction model

## Discussion

This study is the first to develop a diagnostic prediction model for IE based on the 2023 Duke-ISCVID criteria. Designed for rapid application across all clinical departments, this model uses five objective variables that do not depend on blood culture results: presence of a cardiac murmur, neutrophil percentage, log-transformed platelet count, presence of pleural effusion, and q-SOFA score ≥ 2.

The model enables early identification of IE as the cause of fever on admission and supports consistent evaluation across institutions and clinicians. In the validation cohort, the model achieved an AUC of 0.859, compared with 0.783 for our previously developed model, which included the following five variables: transfer by ambulance, presence of a cardiac murmur, presence of pleural effusion, neutrophil percentage, and platelet count [5]. Its performance is also comparable to that of the CREED score (AUC 0.881), which currently represents the highest-performing model among the three existing prediction models that do not rely on blood culture results [5, 12–15]. Given its novelty, practicality, and strong performance, the model constitutes a meaningful contribution to the diagnosis of IE.

In our study, a q-SOFA score ≥ 2 was identified as a significant predictor in the diagnostic model. q-SOFA is a screening tool for sepsis, comprising three clinical parameters: altered consciousness, systolic blood pressure ≤ 100 mmHg, and respiratory rate ≥ 22 breaths/min [18]. Sepsis is defined as life-threatening organ dysfunction caused by a dysregulated host response to infection; patients with a q-SOFA score ≥ 2 are considered at high risk [18]. IE is a bloodstream infection caused by bacteria, and can progress to sepsis and multi-organ failure [21]. A higher q-SOFA score has been associated with increased in-hospital mortality among patients with IE [22, 23]. In our validation cohort, patients with IE had higher LDH, BUN, and creatinine levels, and a higher frequency of pleural effusion than patients without IE, suggesting more frequent organ dysfunction. These findings support the clinical relevance of q-SOFA and reinforce the inclusion of a q-SOFA score ≥ 2 as a useful predictor in the diagnostic model for IE.

The higher AUC of the present model, compared with our previous model, may result from replacing “transfer by ambulance” with “q-SOFA score ≥ 2” and from identifying pleural effusion as a significant predictor. Pleural effusion is a marker of clinical severity and has been associated with increased mortality; it may arise from organ dysfunction, malignancy, infection, or inflammatory conditions [24–27]. Although both “transfer by ambulance” and “q-SOFA score ≥ 2” indicate disease severity, transfer status may vary across institutions and populations, whereas q-SOFA is a standardized, objective measure [16, 18, 22, 23]. In our previous model, the inclusion of “transfer by ambulance” may have obscured the prognostic value of pleural effusion [5]. In this study, the inclusion of q-SOFA, a more objective, consistent severity indicator, may have clarified the relationship between pleural effusion and disease severity, thereby enhancing its diagnostic significance in the model.

### Limitations

This study has some limitations. First, the diagnostic prediction model was developed from a retrospective cohort at a single center in Japan and validated using cohorts from four tertiary-care hospitals in Japan. Because some patients with IE may initially seek care in non-tertiary institutions, validation in such settings is needed to assess generalizability. Second, participant identification was based on ICD-10 codes. Patients with undiagnosed fever who were not assigned the R50.9 code, and patients with IE who were not assigned the I33.0 code because of coding errors or an alternative primary diagnosis, may have been inadvertently excluded. Third, patients with R50.9 who did not undergo urinalysis or chest radiography were excluded from the analysis. Therefore, some patients with IE may have been inadvertently excluded. Fourth, this retrospective study could not reproduce and validate the previously published U.S. and Italian models [12–14], as several key predictors used in these models were not collected in our dataset. Finally, the clinical characteristics of patients with IE may vary by country. For example, in the United States, a substantial proportion of patients with IE are linked to intravenous drug use, and affected patients tend to be younger (typically in their 30s to 40s) [12, 13, 28]. Accordingly, the distribution of causative pathogens and the prevalence of underlying conditions may differ from those observed in the Japanese cohort.

## Conclusions

In this study, we developed a diagnostic prediction model for definite IE according to the 2023 Duke-ISCVID criteria. The model incorporates five consistent, easily obtainable variables applicable across hospital settings: presence of a cardiac murmur, presence of pleural effusion, neutrophil percentage, log-transformed platelet count, and q-SOFA score ≥ 2. The model demonstrated good discrimination and calibration. Further multicenter validation in community hospitals, where patients with IE may also present, would enhance generalizability.

## Supplementary Information

Supplementary Material 1.

## Data Availability

The datasets analysed during the current study are available from the corresponding author on reasonable request. The study protocol will be available via the UMIN website (www.umin.ac.jp; UMIN ID: UMIN000058353).

## Abbreviations

AUC: Area under the curve
BUN: Blood urea nitrogen
CI: Confidence interval
CT: Computed tomography
ICD-10: International Statistical Classification of Diseases and Related Health Problems-10th Revision
IE: Infective endocarditis
IQRs: Interquartile ranges
ISCVID: International society for cardiovascular infectious diseases
LASSO: Least Absolute Shrinkage and Selection Operator
LDH: Lactate dehydrogenase
MRI: Magnetic resonance imaging
NPV: Negative predictive value
OR: Odds ratio
PPV: Positive predictive value
q-SOFA: The quick sequential [sepsis-related] organ failure assessment
SIRS: The systemic inflammatory response syndrome
UMIN: University Hospital Medical Information Network
