# Supplementary material for: A new diagnostic prediction model for infective endocarditis based on the 2023 duke–international society for cardiovascular infectious disease criteria: a multicenter observational study
Source: BMC Cardiovasc Disord. 2026 Mar 16;26:349. doi: 10.1186/s12872-026-05742-8 (PMC13104256; doi:10.1186/s12872-026-05742-8)
Supplement: Supplementary file 1 — Supplementary Material 1. [file 12872_2026_5742_MOESM1_ESM.docx]

**Supplementary Material Table 1. Survey items**

| **Survey items** |  |
| --- | --- |
| Patient characteristics | Age, sex, duration of hospital stays, mortality at discharge, 30-day mortality after admission, in-hospital mortality, transfer by ambulance, administration of antibiotics before obtaining blood culture (except long-term administered agents) |
| Past history | Chronic skin disease, chronic kidney disease, diabetes mellitus, malignancy, use of steroid, use of immunosuppressants, infective endocarditis. |
| Vital signs^†^ | Pulse rate, respiratory rate, presence or absence of acute respiratory failure,  presence or absence of altered consciousness, q-SOFA score, SIRS score |
| Physical examinations^†^ | Cardiac murmur |
| Radiological imaging findings^†^ | Pulmonary edema, pleural effusion |
| Laboratory findings^†^ | White blood cell count, neutrophil percentage, platelet count, albumin level,  total- bilirubin level, lactate dehydrogenase, blood urea nitrogen level,  creatinine level, C-reactive protein level |
| Required elements for evaluation according to the 2023-Duke ISCVID | Detected organisms in blood cultures, the number of positive blood cultures, Urinalysis (presence or absence of proteinuria, hematuria, fragmented red blood cells, pathological casts)  The presence or absence of the following items: fulfillment of major and minor criteria for blood cultures; fulfillment of major echocardiographic criteria; Osler's nodes; Janeway lesions; petechiae on the palpebral conjunctiva; Roth spots, Fever ≥ 38°C; underlying heart disease; valvular regurgitation; mitral valve prolapse; prosthetic valve; intracardiac prosthetic material; mycotic aneurysm; embolic cerebral infarction; intracranial hemorrhage; pulmonary infarction; pulmonary embolism; splenic infarction; renal infarction; hepatic infarction; mesenteric artery infarction; performing of cranial CT, contrast-enhanced chest and abdominal CT, and brain MRI; ^18^F-FDG PET-CT uptake in prosthetic valves or devices; and microbial detection and histopathological findings of active infective endocarditis in cardiac valves or intravascular prosthetic material. |

2023-Duke ISCVID, international society for cardiovascular infectious disease criteria for infective endocarditis; q-SOFA, quick sequential [sepsis-related] organ failure assessment; SIRS, systemic inflammatory response syndrome; CT, computed tomography; MRI, magnetic resonance imaging; ^18^F-FDG PET-CT, fluorine-18 fluorodeoxyglucose positron emission tomography–computed tomography

†: On admission

**Supplementary Material Method 1. Implementation of the LASSO regression analysis**

An L1-penalized logistic regression model (LASSO) was fitted using the glmnet engine within the caret framework. All candidate predictors were centered and scaled before model fitting [caret preProcess =c("center","scale")]. The LASSO penalty parameter (λ) was tuned with the mixing parameter fixed at α = 1.0. Candidate λ values were evaluated on a logarithmic grid from 10^-1^ to 10^-10^ [caret tuneGrid =expand.grid(alpha = 1, lambda = 10^-1:10^)]. Model performance for each λ was assessed using 10-fold cross-validation [trainControl(method ="cv", number = 10)], with class probabilities estimated within each fold (classProbs = TRUE). The tuning objective was to maximize the area under the receiver operating characteristic curve (AUC; caret metric ="ROC" with twoClassSummary), rather than to minimize classification error. A fixed random seed [set.seed(2025)] was specified to ensure reproducibility. The final model was refitted on the full training dataset using the λ value that achieved the highest cross-validated AUC. Predictors with non-zero coefficients in this final model were used as the selected variables.

**Supplementary Material Figure 1. Enrollment flow diagram for the development and validation cohorts**

(A) Derivation cohort


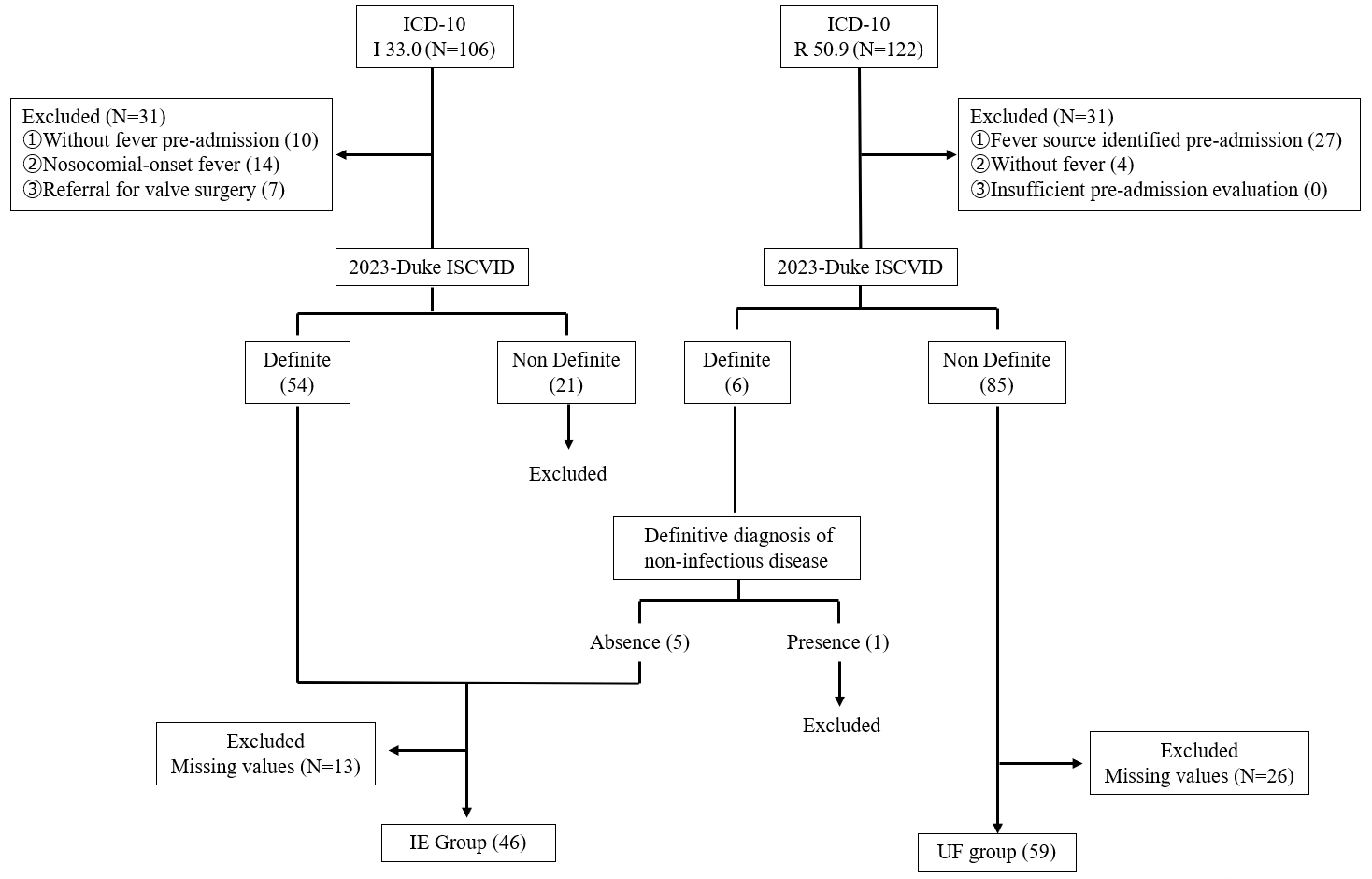


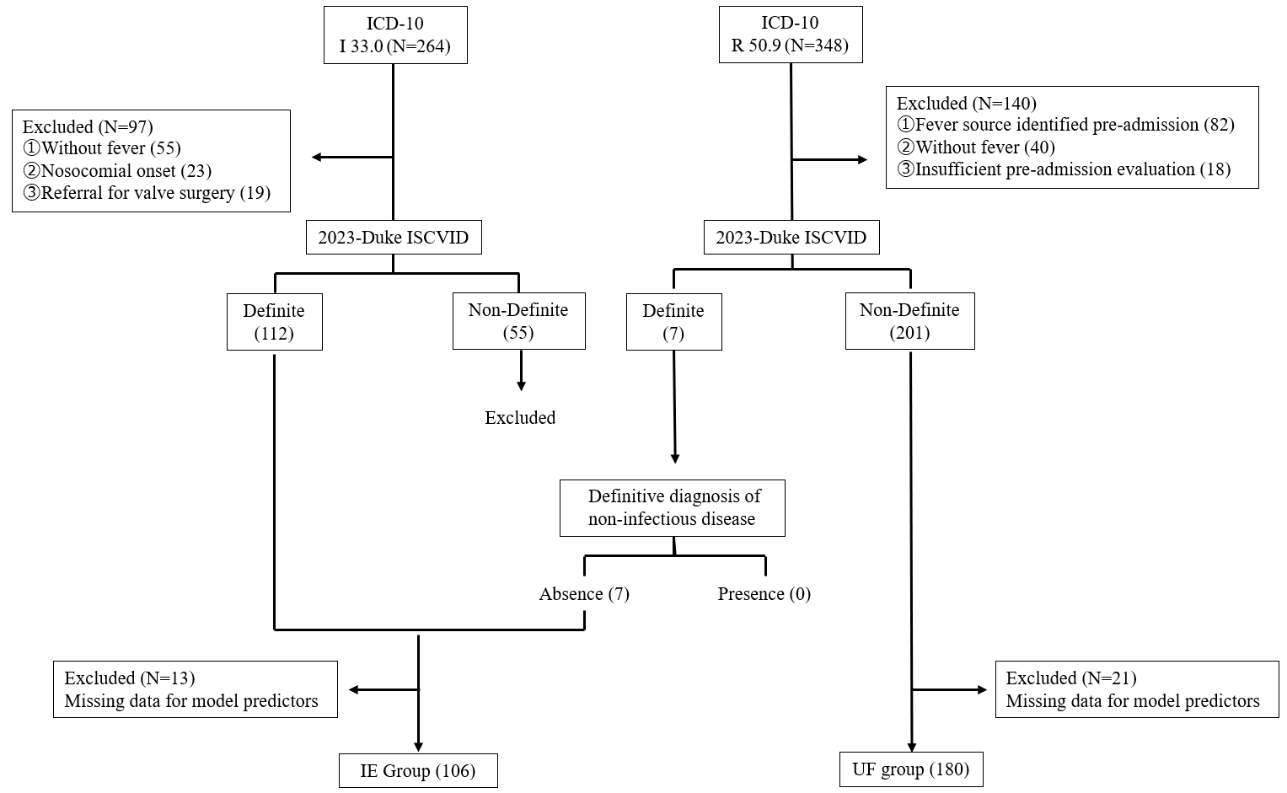
(B) Validation cohort

ICD-10, International Statistical Classification of Diseases and Related Health Problems-10th Revision; 2023-Duke ISCVID, international society for cardiovascular infectious disease criteria for infective endocarditis; IE, infective endocarditis; UF, undiagnosed fever.

**Supplementary Material Table 2. Distribution of causative diseases in the undiagnosed fever group in the derivation and validation cohorts**

| **Derivation cohort** | |
| --- | --- |
| Infectious diseases  (N=24) | Clostridium difficile infection (4), vertebral osteomyelitis (3), aspiration pneumonia (2), herpesvirus meningitis (2), rickettsia infection (2), acute cholangitis (1), acute focal bacterial nephritis (1), acute pyelonephritis (1), bacterial pneumonia (1), extradural abscess (1), fungemia (1), ileitis (1), pharyngeal tonsillitis (1), pyogenic arthritis (1), viral infection (1), viral pericarditis (1) |
| Non-infectious inflammatory diseases  (N=12) | Granulomatosis with polyangiitis (3), incomplete Behcet's disease (2), adult-onset Still’s disease (1), familial Mediterranean fever (1), histiocytic necrotizing lymphadenitis (1), microscopic polyangiitis (1), pseudogout (1), systemic lupus erythematosus (1), ulcerative colitis (1) |
| Malignancies  (N=9) | Diffuse large B-cell lymphoma (1), Hodgkin lymphoma (1), ileal gastrointestinal stromal tumor (1), intravascular large B-cell lymphoma (1), malignant lymphoma (1), peripheral T-cell lymphoma (1), peritoneal cancer (1), systemic metastasis of ovarian cancer (1), urethral cancer (1) |
| Others^§^  (N=14) | Fever of unknown origin (6), chronic arthritis (1), drug rash (1), idiopathic eosinophilic syndrome (1), malignant syndrome (1), mesenteric panniculitis (1), multiple liver cysts (1), non-occlusive mesenteric ischemia (1), subacute arthritis (1) |
| **Validation cohort** | |
| Infectious diseases  (N=66) | Acute pyelonephritis (9), bacteremia (9), viral infection (8), bacterial infection (5), acute pneumonia (3), acute prostatitis (3), urinary tract infection (3), aseptic meningitis (2), aspiration pneumonia (2), iliopsoas muscle abscess (2), catheter sepsis (1), bacterial meningitis (1), bacterial pneumonia (1), bronchial pneumonia (1), candida septicemia (1), cytomegalovirus infection (1), infection of artificial blood vessel after transplantation (1), infected aortic aneurysm (1), influenza virus infection (1), listeria meningitis (1), odontogenic maxillary sinusitis (1), retropharyngeal abscess (1), salmonella enteritis (1), septic shock (1), sinusitis (1), Epstein–Barr viral infection (1), Epstein–Barr viral hepatitis (1), miliary tuberculosis (1), non-tuberculous mycobacteriosis (1), pelvic connective tissue inflammation (1) |
| Non-infectious inflammatory diseases  (N=26) | Rheumatoid arthritis (7), adult-onset Still’s disease (5), Sjogren’s syndrome (2), ulcerative colitis (2), hemophagocytic lymphohistiocytosis (2), aorta inflammation (1), familial Mediterranean fever (1), histiocytic necrotizing lymphadenitis (1), polymyalgia rheumatica syndrome (1), pseudogout (1), sarcoidosis (1), systemic lupus erythematosus (1), ANCA-associated vasculitis (1) |
| Malignancies  (N=11) | Acute myelocytic leukemia (2), malignant lymphoma (2), paraneoplastic syndrome (2), biliary tract cancer (1), Castleman's disease (1), epipharyngeal cancer (1), intravascular large B-cell lymphoma (1), multiple myeloma (1) |
| Others^§^  (N=77) | Fever of unknown origin (67), drug rash (2), acute hepatitis (1), catatonic schizophrenia (1), chronic chemical bronchitis (1), chronic gingivitis (1), chronic thromboembolic pulmonary hypertension (1), drug hypersensitivity (1), hypertrophic cardiomyopathy (1), Parkinson’s disease (1) |

Each disease is presented as “disease name (number)”.

§: includes cases whose final diagnosis was fever of unknown origin and conditions that could not be classified into infectious diseases, non‑infectious inflammatory diseases, or malignancies.
